# Mites of the genus *Neharpyrhynchus* Fain (Acariformes, Harpirhynchidae) from Neotropical birds

**DOI:** 10.3897/zookeys.89.974

**Published:** 2011-04-11

**Authors:** Bochkov Andre V., Literak Ivan

**Affiliations:** 1 *Zoological Institute, Russian Academy of Sciences, Universitetskaya Embankment 1, 199034 Saint Petersburg, Russia*; 2 *Museum of Zoology, University of Michigan, 1109 Geddes Avenue, Ann Arbor, Michigan 48109-1079, U.S.A.*; 3 *Department of Biology and Wildlife Disease, Faculty of Veterinary Hygiene and Ecology, University of Veterinary and Pharmaceutical Sciences, Palackeho 1-3, 612 42 Brno, Czech Republic*

**Keywords:** Acariformes, Harpirhynchidae, *Neharpyrhynchus*, systematics, birds, parasites

## Abstract

Three new species of parasitic mites of the genus Neharpyrhynchus Fain (Acariformes, Harpirhynchidae) are described from Neotropical birds: Neharpyrhynchus chlorospingus **sp. n.** from Chlorospingus pileatus (Passeriformes, Emberizidae) from Costa Rica, Neharpyrhynchus mironovi **sp. n.** from Dacnys cayana (Passeriformes, Thraupidae) and Neharpyrhynchus tangara **sp. n.** from Tangara cayana (Thraupidae) both from Brazil. Neharpyrhynchus trochilinus (Fain) is recorded from 3 new host species of the family Trochilidae (Apodiformes), Panterpe insignis and Eugenes fulgens from Costa Rica, and Amazilia lactea from Brazil. Emended diagnosis of the genus and a key to species are provided; all records of Neharpyrhynchus species are summarized.

## Introduction

Mites of the genus Neharpyrhynchus Fain (Acariformes, Harpirhynchidae) are permanent and highly specialized parasites of birds, as is the case for all other representatives of the subfamily Harpirhynchinae (Bochkov 2008). The subgenus Neharpyrhynchus Fain was established by Fain (1972) in the genus Harpirhynchus Mégnin. Later, Fain (1995) proposed full generic status for Neharpyrhynchus and simultaneously revised this genus, which included five species at that time. The life-cycle of these mites was described by Moss et al. (1968) as exemplified by Neharpyrhynchus novoplumaris Moss et al. The last revision of the genus Neharpyrhynchus was recently provided by Martinu et al. (2008). To date this genus includes 11 species belonging to five species groups established in that revision: *baile* (3 species)*, hippolae* (3 species), *pilirostris* (1 species), *plumaris* (3 species),and *squamiferus* (1 species). In our opinion, however, there are no characters discriminating the *pilirostris* and *hippolae* species groups. Moreover, such differential characters were not provided even by Martinu et al. (2008) and in their key, Neharpyrhynchus pilirostris is placed in the same couplet with Neharpyrhynchus pari, a species from the group *hippolae*. We, therefore, include all species of the *hippolae* group in the *pilirostris* group.

Most species of the genus are known from European passerines and only two species are known from Neotropical birds, Neharpyrhynchus baile Bochkov et al. from Turdus leucomelas (Passeriformes, Turdidae) (Bochkov et al. 2007) andNeharpyrhynchus trochilinus (Fain) from hummingbirds (Fain 1972, 1995). In this paper, we describe three new species from Neotropical birds and provide new records for Neharpyrhynchus trochilinus. Additionally, an emended diagnosis of the genus and a key to its species are given. The diagnostic characters of species groups we recognize in the genus Neharpyrhynchus and all records of these mites are given in Tables 1 and 2, respectively.

**Table 1.** Subdivision of the genus Neharpyrhynchus Fain on species groups. Characters:**1** Setae *vF*: smooth (0), serrate (1) **2** Setae *3a*: present (0), absent (1) **3** Number of articulated segments of leg I: 4 (0), 2 (1) **4** Number of articulated segments of legs II: 4 (0), 2 (1) **5** Number of articulated segments of legs IV: 2 (0), 1 (1) **6** Ornamentation of anterior region of propodsoma: absent or almost absent (0), present (1).

Groups	Characters						Species
		1	2	3	4	5		6
baile	1	0	0	0	1	0	Neharpyrhynchus baile Bochkov et al., Neharpyrhynchus bochkovi Martinu et al., Neharpyrhynchus trochilinus (Fain)
plumaris	0	0	1	1	1	1	Neharpyrhynchus chlorospingus sp. n., Neharpyrhynchus novoplumaris (Moss et al.), Neharpyrhynchus plumaris (Fritsch), Neharpyrhynchus spinus Martinu et al.
pilirostris	0	1	1	1	1	1	Neharpyrhynchus hippolae Bochkov, Neharpyrhynchus mironovi sp. n., Neharpyrhynchus pari Martinu et al., Neharpyrhynchus pilirostris (Berlese & Trouessart), Neharpyrhynchus schoenobaenus Martinu et al., Neharpyrhynchus tangara sp. n.
squamiferus	0	0	0	1	0	1	Neharpyrhynchus squamiferus (Fain)

**Table 2.** Distribution of Neharpyrhynchus spp. on hosts. **“** - The same data as previous; * - type host; **&** - probably accidental record or wrong determination; **@** - originally determined as Neharpyrhynchus plumaris Fritsch, 1954; **$** - originally determined as Neharpyrhynchus novoplumaris Moss et al., 1968.

Mite species	Host species	Host family and order	Locality	Reference
Neharpyrhynchus baile Bochkov et al., 2007	*Turdus leucomelas Vieillot	Turdidae (Passeriformes)	Brazil (Mato Grosso do Sul)	Bochkov et al. (2007)
Neharpyrhynchus bochkovi Martinu et al., 2008	*Turdus merula Linnaeus	Turdidae (Passeriformes)	Czech Republic	Martinu et al. (2008)
Neharpyrhynchus chlorospingus sp. n.	*Chlorospingus pileatus Salvin	Emberizidae (Passeriformes)	Costa Rica	Present paper
Neharpyrhynchus hippolae Bochkov, 2000	*Hippolais icterina (Vieillot)	Sylviidae (Passeriformes)	Russia (Novgorod Prov.)	Bochkov (2000)
Neharpyrhynchus mironovi sp. n.	*Dacnis cayana (Linnaeus)	Thraupidae (Passeriformes)	Brazil (Minas Gerais)	Present paper
Neharpyrhynchus novoplumaris (Moss et al., 1968)	*Certhia familiaris Linnaeus	Certhiidae (Passeriformes)	USA (California)	Moss et al. (1968)
“	Cardinalis cardinalis (Linnaeus)	Cardinalidae (Passeriformes)	USA (Maryland, Nebraska)	Moss et al. (1968)
“	&Campylorhynchus brunneicapillus (Lafresnaye)	Troglodytidae (Passeriformes)	USA (?)	Moss (1979)
“	&Spizella passerina (Bechstein)	Emberizidae (Passeriformes)	USA (?)	Moss (1979)
“	&Amphispiza bilineata (Cassin)	Emberizidae (Passeriformes)	USA (?)	Moss (1979)
“	&Melozone fusca (Swainson)	Emberizidae (Passeriformes)	USA (?)	Moss (1979)
Neharpyrhynchus pari Martinu et al., 2008	*Parus major (Linnaeus)	Paridae (Passeriformes)	Czech Republic	Martinu et al. (2008)
“	Periparus ater (Linnaeus)	Paridae (Passeriformes)	Czech Republic	Martinu et al. (2008)
“	@“	“	unknown	Moss (1979)
“	Cyanistes caeruleus (Linnaeus)	Paridae (Passeriformes)	Czech Republic	Martinu et al. (2008)
“	Poecile montanus (Baldenstein)	Paridae (Passeriformes)	Czech Republic	Martinu et al. (2008)
“	Poecile palustris (Linnaeus)	Paridae (Passeriformes)	Czech Republic	Martinu et al. (2008)
“	@“	Paridae (Passeriformes)	unknown	Moss (1979)
“	$Baeolophus bicolor (Linnaeus)	Paridae (Passeriformes)	USA (?)	Moss (1979)
Neharpyrhynchus pilirostris (Berlese & Trouessart, 1889)	*Passer domesticus (Linnaeus)	Passeridae (Passeriformes)	France	Berlese and Trouessart (1889)
“	“	“	Germany	Fritsch (1954); Lawrence (1959)
“	“	“	Czech Republic	Martinu et al. (2008)
“	“	“	South Africa	Lawrence (1959)
“	“	“	USA (Kansas)	Fain (1995)
“	&Aegithalos caudatus (Linnaeus)	Aegithalidae (Passeriformes)	unknown	Moss (1979)
Neharpyrhynchus plumaris (Fritsch, 1954)	*Fringilla coelebs (Linnaeus)	Fringillidae (Passeriformes)	Germany	Fritsch (1954)
“	“	“	Czech Republic	Martinu et al. (2008)
“	“	“	Russia (Novgorod Prov.)	Bochkov (2000)
“	&Muscicapa striata (Pallas)	Muscicapidae (Passeriformes)	Germany	Fritsch (1954)
“	&Aythya ferina (Linnaeus)	Anatidae (Anseriformes)	Germany	Fritsch (1954)
Neharpyrhynchus schoenobaenus Martinu et al., 2008	*Acrocephalus schoenobaenus (Linnaeus)	Sylviidae (Passeriformes)	Czech Republic	Martinu et al. (2008)
Neharpyrhynchus spinus Martinu et al., 2008	*Spinus spinus (Linnaeus)	Fringillidae (Passeriformes)	Czech Republic	Martinu et al. (2008)
“	@Carduelis cannabina (Linnaeus)	Fringillidae (Passeriformes)	Germany	Fritsch (1954)
Neharpyrhynchus tangara sp. n.	*Tangara cayana	Thraupidae (Passeriformes)	Brazil (Minas Gerais)	Present paper
Neharpyrhynchus trochilinus (Fain, 1972)	Hummingbird	Trochilidae (Apodiformes)	South America (?)	(Fain (1972, 1995)
“	Chrysolampis mosquitus (Linnaeus)	Trochilidae (Apodiformes)	South America (?)	Fain (1995)
“	Panterpe insignis Cabanis & Heine	Trochilidae (Apodiformes)	Costa Rica	Present paper
“	Eugenes fulgens (Swainson)	Trochilidae (Apodiformes)	Costa Rica	Present paper
“	Amazilia lactea (Lesson)	Trochilidae (Apodiformes)	Brazil (Minas Gerais)	Present paper
Neharpyrhynchus squamiferus (Fain, 1972)	*Temenuchus pagodarum (Gmelin)	Sturnidae (Passeriformes)	India (?)	(Fain (1972, 1995)

## Material and methods

Birds were examined by naked eye for the presence of harpirhynchids and released back to the wild. S.V. Mironov and I. Literak examined birds in the field. Mites were cleared in lactophenol and mounted in Hoyer’s medium. Specimens were studied using a Leica microscope under Nomarsky interference-contrast-phase (DIC) optics. Drawings were made with a camera lucida, and measurements were taken using a calibrated ocular micrometer. Drawings were made by A. V. Bochkov. In the species description, names of the leg and idiosomal setae follow (Grandjean (1939, 1944) as adapted by Kethley (1990). Names of the palpal setae follow Grandjean (1946) as adapted by Bochkov (2008). All measurements are given in micrometers (μm) and were made according to the standard method (Bochkov et al. 2007): body length = maximum length of the body up to the anterior extremity of the palpal tibia; body width = maximum width taken at whatever level it occurs; gnathosomal length = length taken ventrally from the gnathosomal base to the anterior extremity of the palpal tibia; gnathosomal width = maximum width taken at whatever level it occurs; length of dorsal shield = maximum length, measured in the median line of the shield; and width of dorsal shield = maximum width taken at whatever level it occurs.

The scientific names of birds follow the checklist of Clements et al. (2010).

**Abbreviations:**

CM #	Ivan Literak field number;

INBio	National Biodiversity Institute (Instituto Nacional de Biodiversidad), Heredia, Costa Rica;

IPCR Institute of Parasitology, Academy of Sciences of the Czech Republic, České Budějovice, Czech Republic

IRSNB	Royal Belgian Institute of Natural Sciences (Institut Royal des Sciences Naturelles de Belgique), Brussels, Belgium;

MZUSP	Zoological Museum of the University of São Paulo (Museu de Zoologia da Universidade de São Paulo), Brazil;

SVM #	S. Mironov field number;

UMMZ	University of Michigan Museum of Zoology, Ann Arbor, USA;

ZISP	Zoological Institute of the Russian Academy of Sciences, Saint Petersburg, Russia;

ZISP AVB #	number in collection of ZISP.

## Systematics

**Family Harpirhynchidae Dubinin**

### Genus Neharpyrhynchus

Fain

#### Type species:

Harpyrhynchus plumaris Fritsch, 1954: 193, figs 11, 12, by original designation

#### Diagnosis.

##### Female.

Subcapitulum bearing setae *n, m*, and *elcp*; palp bearing setae *vF, dF, dG, l”G, dT, l”T, l”Ta*. Setae *vF* smooth or serrate, setae (=palpalae) *dF, dG*, and *l”G* grouped together, strongly thickened and roughly barbed. Membranous part of palpal tarsi bearing 2 microspurs. Idiosoma saccate. Anterior part of propodonotum sclerotized (see remark below); this sclerotized area smooth or sculptured. Dorsal shield distinctly developed, without ornamentation or finely ornamented. Idiosomal setae: *vi, ve*, and *si* set close to each other in anterior part of propodosoma, barbed filiform; *se* and *c2* situated distinctly far from *si*; *h1* – whip-like; *1a*, *1b* – fine, smooth filiform; setae *3a* present or absent; setae *scx* and *ag* absent. Legs I–II moderately reduced, with distinct basal lobes; their pretarsi with pair of angled claws and ciliated empodium each. Leg I with 2–4 articulated segments. Tarsus I with 8 setae (*tc’, tc”, p’, p”, a’, a”, u’, u”*) and 1 straight solenidion *ω1*I; tibia I with 5 setae (*d, l’, l”, v’, v”*), two other proximal segments (if present) devoid of setae. Leg II with 2–4 articulated segments. Tarsus II with 7 setae (*tc’, tc”, p”, a’, a”, u’, u”*) and 1 straight solenidion *ω1*II; tibia II with 5 setae (*d, l’, l”, v’, v”*), two other proximal segments (if present) devoid of setae. Posterior legs III and IV bearing 4–6 setae each; legs III with 1 segment, legs IV with 1 or more rarely with 2 segments.

##### Male.

Gnathosoma as in female. Idiosoma rhomboid in outline. Anterior sclerotized area of propodosoma absent. Dorsal shield well developed, occupying most part of dorsal idiosomal surface. Genital opening situated in middle part of dorsal shield. Genital setae 3 pairs. Penis originating behind genital opening. Situations of dorsal idiosomal setae typical for subfamily. Setae *3a* present. Legs I and II well developed, without basal lobes, with 5 articulate segments each. Setation of tibia and tarsi as in females, three other proximal segments with setae. Legs III with two segments, both bearing setae; legs IV with one segment.

#### Species included:

Neharpyrhynchus baile Bochkov et al., Neharpyrhynchus bochkovi Martinu et al., Neharpyrhynchus chlorospingus sp. n., Neharpyrhynchus hippolae Bochkov, Neharpyrhynchus mironovi sp. n., Neharpyrhynchus novoplumaris (Moss et al.), Neharpyrhynchus pari Martinu et al., Neharpyrhynchus pilirostris (Berlese & Trouessart), Neharpyrhynchus plumaris (Fritsch), Neharpyrhynchus schoenobaenus Martinu et al., Neharpyrhynchus spinus Martinu et al., Neharpyrhynchus squamiferus (Fain), Neharpyrhynchus tangara sp. n., and Neharpyrhynchus trochilinus (Fain).

#### Hosts:

Passeriformes: Aegithalidae, Cardinalidae, Certhiidae, Emberizidae, Fringillidae, Muscicapidae, Paridae, Passeridae, Sturnidae, Sylviidae, Thraupidae, Troglodytidae, Turdidae; Apodiformes: Trochilidae.

#### Remarks.

**Figure 1.**
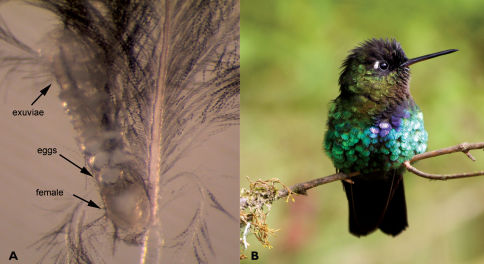
**A** Neharpyrhynchus chlorospingus sp. n., gravid female attached to host feather (photographed by A. V. Bochkov) **B** Panterpe insignis (Trochilidae) – host of Neharpyrhynchus trochilinus (Fain) (photographed by Z. Literakova).

The sclerotized area on the anterior part of the propodonotum was incorrectly named as the propodosomal (=propodonotal) shield by Martinu et al. (2008). In Harpirhynchidae, actually, the true propodonotal shield is fused with the hysteronotal shield or its remnants to form a common large shield, which can be referred to as the dorsal shield (Bochkov 2008). The sclerotized area in the anterior part of the propodosoma situated anterior to the dorsal shield is formed *de novo* and probably helps to fix the subcapitulum when the female attaches to a feather (Fig. 1).

### Neharpyrhynchus chlorospingus

Bochkov & Literak sp. n.

urn:lsid:zoobank.org:act:60C5B820-F280-4D12-9C80-BE5B9D9B2BCD

Figs 2 3 6A 

#### Type material.

Female holotype (ZISP H-T-8, AVB 10-1210-001) and 4 female paratypes (ZISP AVB 10-1210-001, 1–4) from Chlorospingus pileatus Salvin (Passeriformes, Emberizidae) [feathers around ear apertures], COSTA RICA: Cerro de la Mueste, 9°34'N, 83°45'W, 13 August 2010, coll. I. Literak et al. (CM 112).

#### Type deposition.

Holotype and 3 paratypes deposited in the ZISP, one paratype in the INBio.

#### Description.

##### Female (holotype).

**Figure 2.**
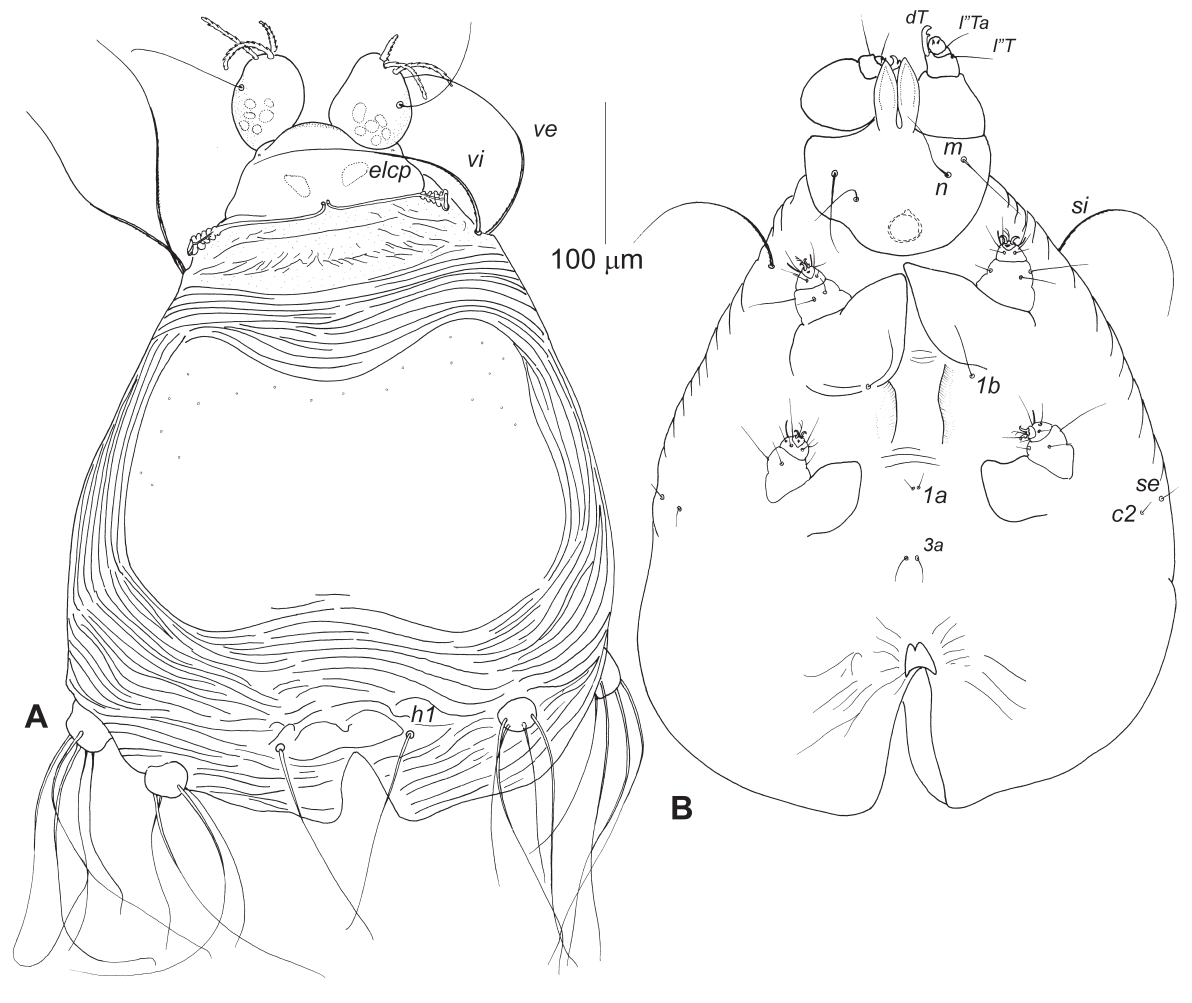
Neharpyrhynchus chlorospingus sp. n., female holotype, **A** dorsal view **B** ventral view.

**Figure 3.**
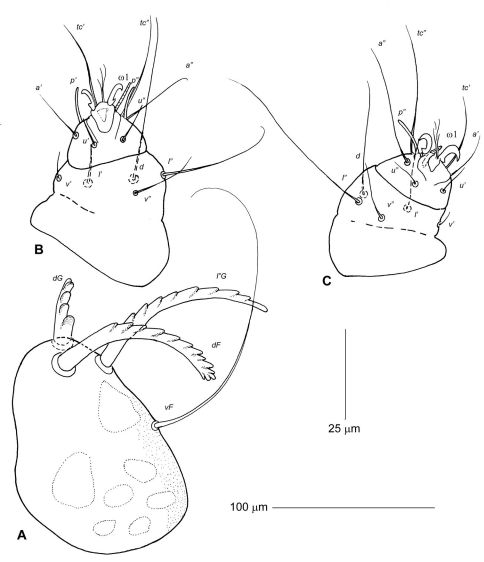
Neharpyrhynchus chlorospingus sp. n., details of female holotype, **A** palp in dorsal view **B** leg I in dorsal view **C** leg II in dorsal view.

Idiosoma, including gnathosoma, 525 long (500–550 in 3 paratypes), 360 wide (350–360) (Fig. 2). Gnathosoma 130 long (130–145), 130 wide (130–140). Palps 50–60 long, moderately inflated dorsally. All palpalae distinctly pectinate (Fig. 3A). Lengths of palpalae: *dF* 35 (35–35), *dG* 20 (20–25), and *l”G* 50 (40–50); *dG* slightly thicker and about 2 times shorter than *dF* and *l”G*. Setae *vF* 100–110 long, smooth. Subcapitulum ventrally with setae *n* and *m*, about 40 and 50 long, respectively. Peritremal branch about 85 long. Idiosoma 425 long (420–440). Anterior region of propodonotum covered by short irregularly situated folds, without scales or tubercles (Fig. 6A). Dorsal shield entire, 165 long in midline (160–170), 300 at maximum width (300–330) (Fig. 2A). Anterior and posterior margins of dorsal shield widely concave. Ventral surface of idiosoma with indistinct transverse striations, without scales or verrucosities (Fig. 2B). Setal lengths: *vi, ve*, and *si* - all distinctly barbed, subequal in length, 150–160; *se, c2*, and *1a* - all smooth, 10–12; *h1* whip-like, 250 (230–260); *1b* smooth, about 40; *3a* present, about 20. Base of legs I with distinctly developed and slightly attenuated fleshy lobe; base of legs II with moderately developed rounded lobe. Leg I with 2 articulated segments (Fig. 3B). Leg II with 2 articulated segments (Fig. 3C). Legs III, IV with one segment, each bearing 4 (more rarely 5) long setae. One ventral seta of leg III and 2 ventral seta of leg IV 100–120 long, about half the length of other setae situated dorsally or dorsoterminally, 200–250 long.

##### Male.

Unknown.

#### Etymology.

The species name is derived from the generic name of the host and is a noun in apposition.

#### Differential diagnosis.

This species belongs to the “*plumaris”* species group including three species, Neharpyrhynchus plumaris (Fritsch), Neharpyrhynchus novoplumaris (Moss et al.), and Neharpyrhynchus spinus Martinu et al. (Martinu et al. 2008). In females of this group, legs I and II consist of the two articulated segments, palpal setae *vF* are smooth, the anterior region of the propodonotum is covered by short irregular striations, and setae *3a* are present. Within this group, it is close to Neharpyrhynchus novoplumaris described from Certhia familiaris Linnaeus (Passeriformes, Certhiidae) [type host] and Cardinalis cardinalis (Linnaeus) (Passeriformes, Cardinalidae) from USA (Moss et al. 1968). In females of both of these species setae *dG* are about half the lenth of *l”G*. In the other two species of the genus, setae *dG* and *l”G* are subequal. Females of Neharpyrhynchus chlorospingus differ from Neharpyrhynchus novoplumaris by the following characters. In Neharpyrhynchus chlorospingus, palpal setae *dF* are slightly shorter than *l”G*, setae *se* and *c2* are about four times shorter than *1b*, the posterior margin of the dorsal shield is widely concave. In Neharpyrhynchus novoplumaris, palpal setae *dF* are slightly longer than *l”G*, setae *se* and *c2* are subequal or only slightly shorter than *1b*, the posterior margin of the dorsal shield is widely convex.

### Neharpyrhynchus mironovi

Bochkov & Literak sp. n.

urn:lsid:zoobank.org:act:07B0EB50-C713-417B-AAA4-0B240A93AA7D

Figs 4 5A–C 6B 

#### Type material.

Female holotype (MZUSP), 20 female paratypes (ZISP AVB 10-1210-002, #1–20) on slides and numerous paratypes preserved in alcohol from Dacnis cayana (Linnaeus) (Passeriformes, Thraupidae) [feathers around ear apertures, back of the head and neck], BRAZIL: Minas Gerais, Belo Horizonte, Nova Lima, Área de Proteção Permanente (Permanent area for protection) do Condomínio Miguelão, 20°07'S, 43°58'W, 8 September 2010, coll. S.V. Mironov et al. (SVM-10-0908-1).

#### Type deposition.

Holotype and 10 paratypes deposited in the MZUSP, 6 paratypes in the ZISP, 2 paratypes in the UMMZ, and 2 paratypes in the IPCR. Alcohol preserved paratypes deposited in the MZUSP and ZISP.

#### Description.

##### Female (holotype).

**Figure 4.**
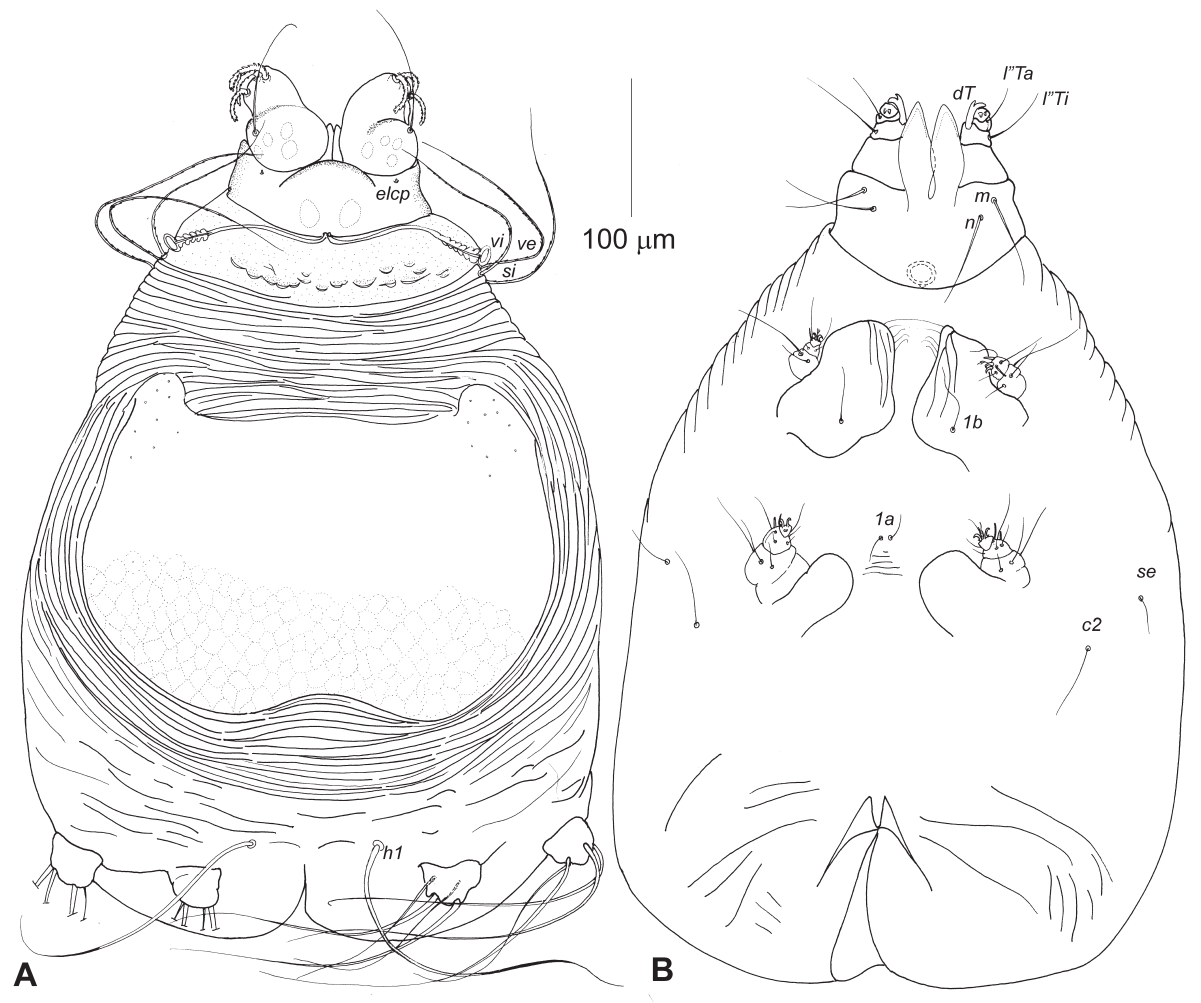
Neharpyrhynchus mironovi sp. n., female holotype, **A** dorsal view **B** ventral view.

**Figure 5.**
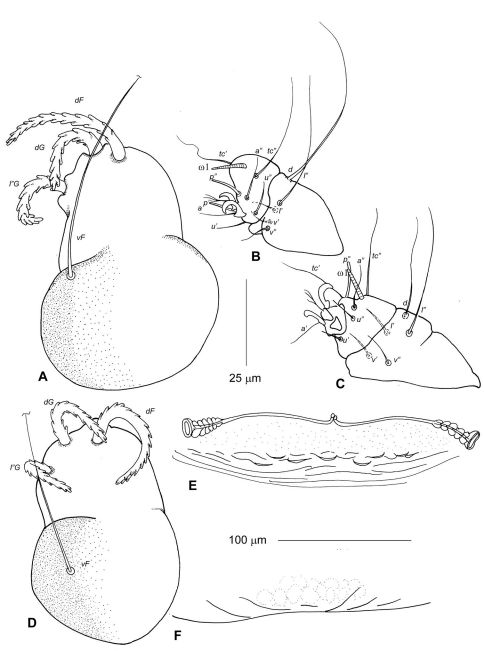
Neharpyrhynchus spp, details of females. Neharpyrhynchus mironovi sp. n., holotype (**A–C**), **A** palp in dorsal view **B** leg I in dorsal view **C** leg II in dorsal view; Neharpyrhynchus tangara sp. n. (**D–F**), **D** palp in dorsal view **E** anterior part of propodonotum **F** posterior margin of dorsal shield. Scale bars: **A–D** = 25 μm; **E** and **F** = 100 μm.

**Figure 6.**
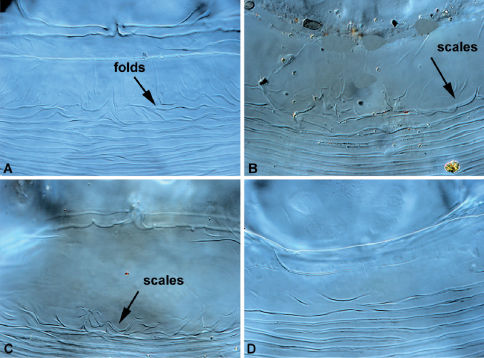
Neharpyrhynchus spp, anterior part of propodonotum, **A** Neharpyrhynchus chlorospingus sp. n. **B** Neharpyrhynchus mironovi sp. n. **C** Neharpyrhynchus tangara sp. n. **D** Neharpyrhynchus trochilinus (Fain).

Idiosoma, including gnathosoma, 675 long (660–680 in 10 paratypes), 425 wide (420–435) (Fig. 4). Gnathosoma 135 long (130–140), 150 wide (140–155). Palps 65–75 long, distinctly inflated dorsally. All palpalae distinctly pectinate (Fig. 5A). Lengths of palpalae: *dF* 40 (38–40), *dG* 30 (28–33), and *l”G* 30 (30–35); *dF* only slightly longer than *dG* and *l”G*. Setae *vF* about 100 long, smooth. Subcapitulum ventrally with setae *n* and *m*, about 80 long. Peritremal branch about 120 long. Idiosoma 525 long (510–530). Anterior region of propodonotum covered by short rounded scales situated irregularly in its posterior half (Fig. 6B). Dorsal shield entire, 200 long in midline (190–200), 350 at maximum width (350–370) (Fig. 4A). Anterior margin of dorsal shield almost straight, with pair of lateral anteriorly directed projections; posterior margin with distinct median concavity. This shield covered by fine rhomboid-like pattern, almost indistinct in anterior half and more clearly discernible in posterior half. Ventral surface of idiosoma with indistinct transverse striations, without scales or verrucosities (Fig. 4B). Setal lengths: *vi, ve*, and *si* - all distinctly barbed, subequal in length, 160–175; *se* and *1a* 12–25*, c2* 50–60- all smooth; *h1* whip-like, 250 (250–280); *1b* smooth, 30–40, *3a* absent. Base of legs I with distinctly developed fleshy lobe partially covering leg segments; base of legs II with moderately developed rounded lobe. Leg I with 2 articulated segments (Fig. 5B). Leg II with 2 articulated segments (Fig. 5C). Legs III, IV with one segment, each bearing 4 long setae. One ventral seta of leg III and 2 ventral seta of leg IV about 150 long, about half the length of other setae situated dorsally or dorsoterminally, 250–300 long.

##### Male.

Unknown.

#### Etymology.

The species is named in honour of the prominent Russian acarologist Dr. Sergey V. Mironov (ZISP).

#### Differential diagnosis.

It is close to species of the group “*pilirostris”*. In all these species, setae *vF* are smooth, only two articulated segments on legs I and II are present, and setae *3a* are absent. Among species of this group, Neharpyrhynchus mironovi is close to Neharpyrhynchus pari by the presence of four setae on leg III and by irregular ornamentation of the anterior part of the propodosoma. The new species differs fromNeharpyrhynchus pari by the following characters. In females of Neharpyrhynchus mironovi, the palps are distinctly inflated dorsally, the ornamentation of the anterior part of the propodonotum is scale-like and present only in the posterior half of this region, setae *c2* are 50–60 long. In Neharpyrhynchus pari, the palps are moderately inflated dorsally, the anterior part of the propodonotum is fully ornamented by verrucosities and setae *c2* are 5–6 long.

### Neharpyrhynchus tangara

Bochkov & Literak sp. n.

urn:lsid:zoobank.org:act:5C1328B7-9A79-445A-AFB6-BC5402DFC36C

Fig. 5D–F 6C 

#### Type material.

Female holotype (MZUSP), 20 female paratypes (ZISP AVB 10-1210-003, 1–20) on slides and numerous paratypes preserved in alcohol from Tangara cayana (Linnaeus) (Passeriformes, Thraupidae) [feathers around ear apertures, back of the head and neck], BRAZIL: Minas Gerais, Belo Horizonte, Nova Lima, Água Limpa, 20°13'S, 43°56'W, 31 August 2010, coll. S.V. Mironov et al. (SVM-10-0831-12).

#### Type deposition.

Holotype and 10 paratypes deposited in the MZUSP, six paratypes in the ZISP, 2 paratypes in the UMMZ, and 2 paratypes in the IPCR. Alcohol preserved paratypes deposited in the MZUSP and ZISP.

#### Description.

##### Female (holotype).

Idiosoma, including gnathosoma, 600 long (600–620 in 10 paratypes), 380 wide (380–400). Gnathosoma 125 long (125–130), 150 wide (145–150). Palps 60 long, distinctly inflated dorsally. All palpalae distinctly pectinate (Fig. 5D). Lengths of palpalae: *dF* 35 (35–40), *dG* 30 (29–33), and *l”G* 29 (30–32); *dF* only slightly longer than *dG* and *l”G*. Setae *vF* about 90 long, smooth. Subcapitulum ventrally with setae *n* and *m*, about 70 long. Peritremal branch about 110 long. Idiosoma saccate, 500 long (500–520). Anterior region of propodonotum covered by short rounded scales situated irregularly in its posterior half (4E, 5C). Dorsal shield entire, 190 long in midline (180–200), 330 at maximum width (330–350). Anterior margin of dorsal shield almost straight with pair of lateral anteriad directed projections; its posterior margin almost straight (4F). This shield covered by fine rhomboid-like pattern, almost indistinct in anterior half and slightly better discernible in posterior half. Ventral surface of idiosoma with indistinct transverse striations, without scales or verrucosities. Setal lengths: *vi, ve*, and *si* - all distinctly barbed, subequal in length, 150–170; *se, c2*,and *1a* 8–12- all smooth; *h1* whip-like, 230 (210–240); *1b* smooth, 30–35, *3a* absent. Legs as in previous species.

##### Male.

Unknown.

#### Etymology.

The species name derives from the generic name of the host and is a noun in apposition.

#### Differential diagnosis.

This new species is closest to Neharpyrhynchus mironovi and differs by the following characters. In females of Neharpyrhynchus tanagra, setae *c2* are 8–12 long, the posterior margin of the dorsal shield is almost straight. In Neharpyrhynchus mironovi, setae *c2* are 50–60 long, the posterior margin of the dorsal shield is widely concave. Both species are collected from the hosts belonging to the family Thraupidae.

### Neharpyrhynchus trochilinus

(Fain, 1972)

Fig. 6D

Harpyrhynchus (Neharpyrhynchus) trochilinus  Fain, 1972: 55.
Neharpyrhynchus trochilinus Fain 1995: 80, figs 17, 18; Bochkov et al. 2007: 38; Martinu et al. 2008: 207, fig. 1 [types in IRSNB].

#### Material examined.

26 females (ZISP AVB 10-1210-004, 1–26) from Panterpe insignis Cabanis & Heine (Apodiformes, Trochilidae) [feathers of neck], COSTA RICA: Cerro de la Mueste, 9°34'N, 83°45'W, 14 August 2010, coll. I. Literak et al. (CM 199); 10 females from same host (ZISP AVB 10-1210-005,1–10) and locality, 11 August 2010, coll. I. Literak et al. (CM 13); 10 females (ZISP AVB 10-1210-006, 1–10) from same host and locality, 13 August 2010, coll. I. Literak et al. (CM 151).

20 females (ZISP AVB 10-1210-007, 1–20) from Eugenes fulgens (Swainson) (Passeriformes, Trochilidae) [feathers of head, chest, and neck], COSTA RICA: Cerro de la Mueste, 9°34'N, 83°45'W, 13 August 2010, coll. I. Literak et al. (CM 152).

27 females (ZISP AVB 10-1210-008, 1–27) from Amazilia lactea (Lesson) (Apodiformes, Trochilidae) [feathers of head and neck], BRAZIL: Minas Gerais, Belo Horizonte, Nova Lima, Área de Proteção Permanente (Permanent area for protection) do Condomínio Miguelão, 20°07'S, 43°58'W, 4 September 2010, coll. S.V. Mironov et al. (SVM-10-0904-1).

#### Hosts and distribution.

This species was briefly diagnosed from both sexes collected from an unidentified species of hummingbird (Trochilidae) that originated from South America (exact locality unknown) and died in the Zoo of Antwerp (Belgium) during its quarantine. Later on, Fain (1995) provided the full description of this species based on the type specimens and newly obtained specimens from Chrysolampis mosquitus (Linnaeus) (Trochilidae) that also originated in South America (without exact locality) and died in the Zoo quarantine. The trochilids, Panterpe insignis, Eugenes fulgens (Costa Rica), and Amazilia lactea (Brazil) are new hosts for this mite species. It is probable, that this species is associated exclusively with hummingbirds and is widely distributed on representatives of this host family.

#### Remarks.

The longitudinally subdivided dorsal shield of this species is an artifact sometimes induced by the mite mounting. In this species, actually, the dorsal shield is entire. It differs from the closely related Neharpyrhynchus baile Bochkov et al. by the following characters. In females of Neharpyrhynchus trochilinus, setae *dF*, *dG*, and *l”G* are subequal, legs III and IV with 5–6 setae each, setae *si* and *se* 25–35 long. In Neharpyrhynchus baile, setae *dF* is about 1.5 times longer than *dG* and *l”G*, legs III and IV as a rule with 4 setae each, setae *si* and *se* are 6–12 long.

## Keys to species of the genus Neharpyrhynchus Fain (females)

(based on Martinu et al. 2008)

**Table:** 

1	Anterior margin of propodonotum without ornamentation or just with few striations. Palpal seta *vF* serrate. Legs II with 4 articulate segments	2
–	Anterior margin of propodonotum ornamented. Palpal seta *vF* smooth. Legs II with 2 articulate segments	4
2	Palpal setae *dF* 1.4–1.5 times longer than *dG* and *l”G*. Setae *si* and *c2* short, 6–12 long. Leg IV normally with 4 setae (rarely with 5 setae)	3
–	Palpal setae *dF*, *dG*, and *l”G* subequal in length. Setae *si* and *c2* relatively long, 25–35 long. Leg IV normally with 5 setae (rarely with 6 setae)	Neharpyrhynchus trochilinus Fain, 1972
3	Lengths of palpal setae *dF*, *dG*, and *l”G* 58–70, 40–49, and 42–53, respectively. Leg III normally with 5 setae (rarely with 4 setae)	Neharpyrhynchus bochkovi Martinu et al., 2008
–	Lengths of palpal setae *dF*, *dG*, and *l”G* 45–54, 30–44, and 30–39, respectively. Leg III normally with 4 setae (rarely with 5 setae)	Neharpyrhynchus baile Bochkov et al., 2007
4	Legs I with 2 segments; legs IV with 1 segment. Scales on ventral surface of idiosoma absent	5
–	Legs I with 4 segments; legs IV with 2 segments. Scales on ventral surface of idiosoma present	Neharpyrhynchus squamiferus (Fain, 1972)
5	Setae *3a* absent	6
–	Setae *3a* present	11
6	Posterior margin of dorsal shield without distinct median suture. Anterior part of propodosoma ornamented by verrucosities, flat cells or widely rounded scales	7
–	Posterior margin of dorsal shield with distinct median suture reaching 1/3 of shield length. Anterior part of propodosoma ornamented by irregular striae (folds)	Neharpyrhynchus schoenobaenus Martinu et al., 2008
7	Palpal femur distinctly inflated dorsally. Anterior part of propodonotum ornamented only in posterior half by widely rounded scales	8
–	Palpal femur moderately inflated dorsally. Anterior part of propodonotum fully ornamented by verrucosities or closed cells	9
8	Setae *c2* 8–12 long. Posterior margin of dorsal shield almost straight	Neharpyrhynchus tanagra sp. n.
–	Setae *c2* 50–60 long. Posterior margin of dorsal shield widely concave	Neharpyrhynchus mironovi sp. n
9	Anterior part of prodorsum covered by irregularly situated verrucosities, not forming transverse rows. Dorsal shield covered by fine ornamentation. Legs III normally with 4–6 setae	10
–	Anterior part of prodorsum covered by verrucosities forming 4–5 transverse rows. Dorsal shield without ornamentation. Legs III with 4 setae	Neharpyrhynchus pilirostris (Berlese & Trouessart, 1889)
10	Dorsal shield 140–165 long, covered by fine longitudinal striation. Legs III with 5–6 setae	Neharpyrhynchus pari Martinu et al., 2008
–	Dorsal shield 165–195 long, covered by fine irregular transverse scale-like striation. Legs III with 4–5 setae	Neharpyrhynchus hippolae Bochkov, 2000
11	Palpal setae *dG* about half the length of *l”G*	12
–	Palpal setae *dG* and *l”G* subequal	13
12	Palpal setae *dF* slightly shorter than *l”G*. Setae *se* and *c2* about 4 times shorter than setae *1b*. Posterior margin of dorsal shield widely concave	Neharpyrhynchus chlorospingus sp. n.
–	Palpal setae *dF* slightly longer than *l”G*. Setae *se* and *c2* subequal or only slightly shorter than setae *1b*. Posterior margin of dorsal shield widely convex	Neharpyrhynchus novoplumaris Moss et al., 1968
13	Anterior part of propodonotum covered by longitudinal striation only in posterior part. Palpal setae *vF* 75–80 long. Dorsal shield 307–345 wide, covered by fine longitudinal scale-like pattern	Neharpyrhynchus plumaris (Fritsch, 1954)
–	Anterior part of propodonotum completely covered by longitudinal striations. Palpal seta *vF* 98–108 long. Dorsal shield 275–280 wide, devoid ornamentation	Neharpyrhynchus spinus Martinu et al., 2008

## Supplementary Material

XML Treatment for Neharpyrhynchus

XML Treatment for Neharpyrhynchus chlorospingus

XML Treatment for Neharpyrhynchus mironovi

XML Treatment for Neharpyrhynchus tangara

XML Treatment for Neharpyrhynchus trochilinus
